# Mitochondrial Function and Microbial Metabolites as Central Regulators of Intestinal Immune Responses and Cancer

**DOI:** 10.3389/fmicb.2022.919424

**Published:** 2022-06-29

**Authors:** Saskia Weber-Stiehl, Lea Järke, Juan Camilo Castrillón-Betancur, Felix Gilbert, Felix Sommer

**Affiliations:** Institute of Clinical Molecular Biology, Kiel University, Kiel, Germany

**Keywords:** mitochondria, microbiota, metabolites, inflammation, cancer

## Abstract

Energy and anabolic metabolism are essential for normal cellular homeostasis but also play an important role in regulating immune responses and cancer development as active immune and cancer cells show an altered metabolic profile. Mitochondria take a prominent position in these metabolic reactions. First, most key energetic reactions take place within or in conjunction with mitochondria. Second, mitochondria react to internal cues from within the cell but also to external cues originating from the microbiota, a vast diversity of associated microorganisms. The impact of the microbiota on host physiology has been largely investigated in the last decade revealing that the microbiota contributes to the extraction of calories from the diet, energy metabolism, maturation of the immune system and cellular differentiation. Thus, changes in the microbiota termed dysbiosis have been associated with disease development including metabolic diseases, inflammation and cancer. Targeting the microbiota to modulate interactions with the mitochondria and cellular metabolism to delay or inhibit disease development and pathogenesis appears an attractive therapeutic approach. Here, we summarize recent advances in developing the therapeutic potential of microbiota-mitochondria interactions for inflammation and cancer.

## Introduction

Mitochondrial bioenergetics and the gut microbiota, the complex assembly of associated microorganisms, are critical for normal cellular and organismal homeostasis. Recently these functional components have also been identified as contributing factors for disease development and severity, for example in intestinal inflammation and colon cancer. Both, mitochondria and the microbiota are metabolically highly active, and we now know that active immune or cancer cells show an altered metabolic profile and that certain metabolic properties are required for their activation or transformation. The microbiota largely impacts mitochondrial metabolism via the production of metabolic substrates or by delivering signaling molecules, which then coordinate a metabolic response. As the composition and activities of the microbiota are highly flexible and can be modulated by dietary components or specific drugs, targeting the microbiota represents a promising approach to tweak the metabolic profiles and to thereby prevent or ameliorate intestinal pathologies. Here, we will summarize our current understanding of the interactions between the microbiota and mitochondria, how these contribute to disease susceptibility and finally how targeting these interactions could be employed for novel therapies for inflammation and cancer.

## Mitochondria – Powerhouse of the Cell

The cytoplasm of mammalian cells harbors hundreds to thousands of mitochondria, an intracellular semi-autonomous organelle. Mitochondria originated by endosymbiosis of bacteria similar to Rickettsiales, which is an order of the class Alphaproteobacteria within the phylum Pseudomonadota. Due to their endosymbiotic origin, mitochondria share several features with prokaryotic organisms including a small genome in the form of mitochondrial DNA (mtDNA), the machinery for protein biosynthesis (i.e., 70S ribosomes), and they have two phospholipid bilayer membranes separating its inner compartment termed matrix from the cytoplasm of the cell (Gray et al., 2001). However, the outer mitochondrial membrane (OMM) contains porins making it permeable for molecules smaller than 5 kDa, whereas the inner mitochondrial membrane (IMM) is impermeable and thus enables the generation of molecular gradients that then can be used to produce energy – the central cellular function of mitochondria. The main fuel for mitochondrial bioenergetics is Acetyl-CoA, which either originates from fatty acid oxidation (FAO) or from pyruvate, that in turn was generated from glucose by cytosolic glycolysis and then imported into the mitochondria. Acetyl-CoA then feeds into the tricarboxylic acid (TCA) cycle (Engelking, 2015), thereby generating NADH and FADH_2_, which power oxidative phosphorylation (OxPhos). Briefly, both NADH and FADH_2_ provide electrons to complexes I and II of the electron transport chain (ETC), to create an electrochemical proton gradient across the IMM, which finally fuels the synthesis of energy in the form adenosine triphosphate (ATP) via OxPhos by ATP synthase (Chance and Williams, 1955; Engelking, 2015). The IMM even creates inward folds called cristae to increase the surface area for ATP production through the ETC (Salisbury-Ruf et al., 2018). In the presence of oxygen, the terminal electron acceptor, a total of 38 ATP molecules are formed from 1 glucose molecule, of which two stem from glycolysis, two from the TCA cycle and the remaining 34 then from the ETC demonstrating the importance of the mitochondria for cellular bioenergetics.

Notably, mitochondria are not restricted to being intracellular but can also be released from the cell. These extracellular encapsulated mitochondria can even be functionally active, regulate important physiological processes such as inflammatory responses or wound healing and potentially contribute to disease development (Boudreau et al., 2014; Joshi et al., 2019; Miliotis et al., 2019; Al Amir Dache et al., 2020; Levoux et al., 2021).

## Mitochondria Play a Key Role in Regulating Cancer and Inflammation

It is now well established that cancer cells of diverse cancer types share certain cellular characteristics – the hallmarks of cancer cells (Hanahan and Weinberg, 2011). Mitochondria are essential for several of these cancer hallmarks, for example, metabolic reprogramming, resisting cell death and tumor-promoting inflammation. Metabolic reprogramming refers to the phenomenon that even in the presence of oxygen cancer cells increase the glucose uptake and rely on “aerobic glycolysis” producing lactate for their growth despite the much lower energetic efficacy (4 instead of 38 ATP per glucose) compared to OxPhos. Although this metabolic switch termed “Warburg-effect” (Warburg et al., 1927; Locasale and Cantley, 2011) was discovered nearly a century ago, its function has not been fully resolved. However, it has been observed to have beneficial effects on the cells, potentially by providing substrates for anabolic metabolism, for example nucleotides and amino acids. Resisting cell death denotes the observation that cancer cells do not respond to the induction of cell death via apoptosis, which can be viewed as “cellular self-destruction.” Apoptosis is a cascade of cysteine-aspartic proteases (caspases) that target vital cellular functions such as DNA replication or protein translation (McBride et al., 2006) and can either be triggered by external signals via activation of cell surface death receptors (extrinsic pathway) or by internal signals such as DNA damage or viral infection (intrinsic pathway) (Ferri and Kroemer, 2001). Mitochondria are critical for the intrinsic apoptosis pathway. They harbor pro- and anti-apoptotic members of the Bcl-2 family along with proteins regulating the bioenergetic metabolite flux and components of the permeability transition pore (mPTP), which altogether modulate the OMM permeabilization (Decaudin et al., 1998; Green and Kroemer, 2004). Upon disruption of the OMM, cytochrome c and Smac/DIABLO proteins that normally reside in the mitochondrial intermembrane space, are released (Saelens et al., 2004) and trigger the formation of the cytosolic apoptosome composed of Apaf-1, procaspase-9 and cytochrome c (Green and Evan, 2002) finally activating caspases and leading to apoptosis. Cancer cells evade apoptosis by accumulating mutations in or downregulating the expression of proapoptotic factors such as p53, a sensor for DNA damage, or the proapoptotic factor BAX (Bcl-2-associated X protein). Similarly, resistance to cell death in cancer cells is often achieved by increasing the expression of antiapoptotic factors (Bcl-2, Bcl-xL) or of survival signals such as insulin-like growth factors (Hanahan and Weinberg, 2011). Tumor-promoting inflammation describes the paradoxical effect that inflammatory responses often enhance carcinogenesis and disease progression, for example, by fostering a proliferative and regenerative response or by releasing DNA-damaging agents such as reactive oxygen species (ROS), that promote mutations and thereby the evolution of cancer cells (Grivennikov et al., 2010; Hanahan and Weinberg, 2011). ROS are generated through various processes during inflammation. First, dedicated ROS-producing enzymes such as NADPH oxidase family members generate ROS either as signaling molecule or as defense response toward pathogenic microorganisms. However, also the bioenergetic functions of mitochondria during OxPhos produce ROS (Li et al., 2004). Leakage of electrons at complexes I and III of the ETC can form superoxide by partial reduction of oxygen. The superoxide dismutases (SOD) 1 and 2 then process the superoxide to hydrogen peroxide, which can finally be detoxified by glutathione peroxidase. Importantly, production of these mitochondrial ROS can be stimulated by microbial antigens such as lipopolysaccharide (LPS) of Gram-negative bacteria and other ligands of Toll-like receptors (TLR) that function in immune signaling to mount an inflammatory response (West et al., 2011).

Interestingly, various types of immune cells also display similar features of metabolic reprogramming as described above for cancer cells. For example, T cells require a switch to aerobic glycolysis much like the “Warburg-effect” to sustain their activation and differentiation (Palmer et al., 2015). In dendritic cells and macrophages, LPS triggers TLR4 signaling and thereby a mitochondrial metabolic reprogramming that is characterized by an enhanced glycolysis, accumulation of succinate and fatty acid synthesis from citrate (Krawczyk et al., 2010; Infantino et al., 2011; O’Neill and Hardie, 2013; Tannahill et al., 2013). The enhanced glycolysis is needed to generate sufficient ATP as during the immune response nitric oxide is generated that inhibits mitochondrial OxPhos (Everts et al., 2012). Therefore, mitochondrial bioenergetics are not only required for normal cellular homeostasis but also play a central role for both immune activation and carcinogenesis.

## Microbiota – We Are Not Alone!

All animals including humans live in close association with a diverse assembly of microorganisms termed microbiota (Clemente et al., 2012) consisting of bacteria, archaea, fungi, viruses but also eukaryotes (Durack and Lynch, 2019). Microorganisms cover essentially all surfaces of the body, but by far the most reside in the gastrointestinal tract. This gut microbiota is dominated by anaerobic bacteria of 500–1,000 different species but only few phyla (Qin et al., 2010). In the past decade it became evident that the microbiota contributes to many if not all aspects of host physiology (Sommer and Bäckhed, 2013). Thus, resident microorganisms form a barrier against pathogenic colonization, instruct proper development and education of the immune system, impact our behavior, and provide essential vitamins, fermentation products and calories through their metabolic capabilities.

## Microbial Metabolites Program Mitochondrial Functions With Consequences for Disease

**FIGURE 1:**
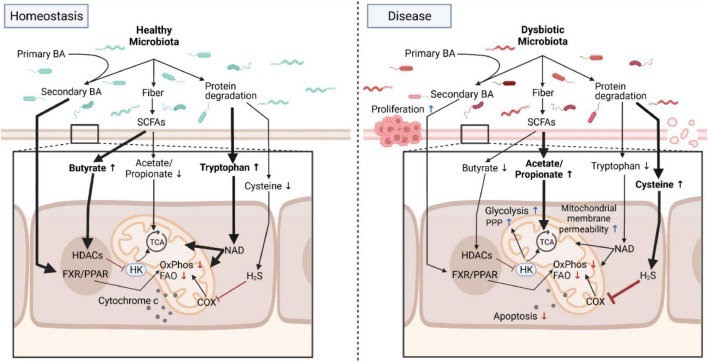
Microbial metabolites program mitochondrial functions in health and disease. Under homeostatic conditions, the healthy microbiota contributes to normal mitochondrial and cellular functions by providing, for example, metabolites that directly feed into mitochondrial bioenergetics or trigger signaling cascades. In diseased states such as chronic intestinal inflammation or cancer, these homeostatic interactions between the dysbiotic microbiota and mitochondria are deregulated and promote a proinflammatory environment fostering carcinogenesis that is characterized by an increase in aerobic glycolysis, a reduction in OxPhos and FAO, changes in mitochondrial membrane permeability and resistance to cell death. Due to its plasticity, interventions targeting the microbiota to restore normal mitochondrial functions are promising novel therapeutic approaches for intestinal inflammation and cancer by counteracting mitochondrial metabolic reprogramming. BA, bile acids; Trp, Tryptophan; SCFA, short-chain fatty acids; FXR/PPAR, farnesoid X receptor/peroxisome proliferators-activated receptor; HK, hexokinase; HDAC, histone deacetylases; TCA, tricarboxylic acid cycle; COX, cyclooxygenase; FAO, fatty acid oxidation; OxPhos, oxidative phosphorylation; PPP, pentose phosphate pathway; NAD, nicotinamide adenine dinucleotide. Created with BioRender.com.

The microbiota interacts with mitochondria by various means including through fermentation products and signaling molecules that directly impact mitochondrial function (Figure 1). Dietary fiber such as resistant starch cannot be metabolized by host enzymes and thus these nutritional components can reach the colon, where they then become available to the colonic microbiota. Members of the microbiota are able to ferment dietary fiber thereby extracting additional calories from the diet and yielding, among others, the short-chain fatty acids (SCFA) acetate, butyrate and propionate. These SCFAs are normally found in a concentration up to 150 mM in a ratio of 3:1:1, respectively (Cummings et al., 1987; den Besten et al., 2013). Among those SCFAs, butyrate appears to be especially important as it serves as the main energy source of intestinal epithelial cells. Butyrate cannot be synthesized by the host but only through the metabolic activities of the microbiota (Louis et al., 2004; Tremaroli and Bäckhed, 2012). In addition to its metabolic potential feeding into mitochondrial bioenergetics, butyrate also (i) engages G protein-coupled receptors GPR41 (FFAR3) (Brown et al., 2003; Vadder et al., 2014), GPR43 (FFAR2) (Brown et al., 2003; Le Poul et al., 2003; Nilsson et al., 2003; Sina et al., 2009) and GPR109a (HCAR2) (Singh et al., 2014; Macia et al., 2015) and (ii) acts on histone deacetylases (HDACs) (Koh et al., 2016), which function as epigenetic regulators, to modulate intracellular signaling pathways and responses. Butyrate is known for many years to have pleiotropic anti-inflammatory properties (Furusawa et al., 2013; Chang et al., 2014; Singh et al., 2014; Koh et al., 2016), yet the exact molecular mechanisms remained elusive. A recent report demonstrated that in T cells, butyrate counteracts mitochondrial alterations that are caused by T cell receptor stimulation (Häselbarth et al., 2020) and enhances mitochondria-dependent apoptosis of activated T cells (Zimmerman et al., 2012), thereby reducing the pro-inflammatory immune responses. Another recent study revealed that in intestinal epithelial cells butyrate engages HDAC8 to downregulate the expression of hexokinase 2, the initial enzyme of glycolysis. In addition, butyrate heavily affects mitochondrial respiration and cell death upon inflammatory challenge (Hinrichsen et al., 2021). Therefore, a microbial metabolite altered colitis susceptibility through metabolic reprogramming, i.e., the modulation of hexokinase and mitochondrial function. Butyrate failed as a therapeutic agent for intestinal inflammation despite encouraging clinical responses due to technical challenges related to its olfactory properties and side-effects (Scheppach et al., 1992; Di Sabatino et al., 2005, 2007). Using more specific inhibitors targeting hexokinase directly or microbiota-focused interventions, i.e., pro-/prebiotic or nutritional supplementations, may prove more promising approaches to treat chronic intestinal inflammation.

As laid out above, butyrate has various beneficial effects alleviating intestinal inflammatory conditions by modulating mitochondrial function. In the context of cancer, however, butyrate seems to play a contrasting role. Elevated levels of butyrate-producing bacteria were identified in colorectal cancer patients and butyrate secreted by these bacteria promoted tumorigenesis (Okumura et al., 2021). On the other hand, *in vitro* experiments using colorectal cancer derived human organoids (Park et al., 2020; Pearce et al., 2020) or colon cancer cell lines revealed anti-tumorigenic effects of butyrate treatment by suppressing proliferation, inducing apoptosis and endoplasmic reticulum stress (Kaiko et al., 2016; Zhang et al., 2016). Butyrate can also by metabolized to β-hydroxybutyrate via the intermediate acetoacetate. A recent study elegantly demonstrated that β-hydroxybutyrate reduced colonic crypt cell proliferation and suppressed intestinal tumor growth via recruiting GPR109A and the transcriptional regulator homeodomain-only protein X (HOPX) (Dmitrieva-Posocco et al., 2022). Furthermore, high β-hydroxybutyrate levels decrease nucleotide binding domain and leucine-rich repeat pyrin 3 (NLPR3) inflammasome formation and antagonize proinflammatory cytokine-triggered mitochondrial dysfunction (Deng et al., 2021).

The pleiotropic effects of butyrate on mitochondrial function and pathogenesis in intestinal inflammation and cancer also extend to other metabolites that are at least partially regulated by the microbiota. In contrast to butyrate, acetate exacerbates colitis (Maslowski et al., 2009), drives hexokinase expression (Hinrichsen et al., 2021) and thereby potentially glycolysis and mitochondrial respiration. To date, there is no consensus concerning the effects on acetate on cancer. In murine intestinal organoids acetate treatment increased levels of Lgr5-positive stem cells and rescued HNF4 (hepatocyte nuclear factor 4) αγ-deficient mice. HNF4 is required for intestinal stem cell renewal and regulates FAO (Chen et al., 2020). In colon cancer cell lines, however, acetate decreased proliferation by targeting the mitochondrial metabolism and reducing glycolysis (Sahuri-Arisoylu et al., 2021). Propionate can promote mitochondrial activity and metabolic reprogramming, which is necessary for propionate-induced expression of MHC class I polypeptide-related sequence A/B (MICA/B) (McCarthy et al., 2018; Høgh et al., 2020). The recognition of MICA is essential for natural killer cells to detect abnormal cells that could transform into cancer cells. Furthermore, in T cells propionate promotes a tolerogenic and anti-inflammatory phenotype by normalizing mitochondrial function through increasing respiration in addition to driving differentiation to regulatory T cells while inhibiting Th1 and Th17 cell differentiation (Duscha et al., 2020).

Branched-chain fatty acids (BCFAs) such as isobutyrate, 2-methylbutyrate, and isovalerate are produced during breakdown of protein under decreased fiber supply. BCFAs elevate acylcarnitine levels indicating an aberrant mitochondrial FAO (Choi et al., 2021) and promote mitochondrial biogenesis by activating the central transcriptional regulators peroxisome proliferators-activated receptor γ (PPARγ) and PPARγ coactivator 1α (PGC-1α) (Ye et al., 2020). Antibiotic therapy leads to downregulation of all FAO enzymes but also changes in microbiota composition and the metabolite pool, especially regarding fatty acids and bile acids (Zarrinpar et al., 2018).

The microbiota also plays an important role in bile acid (BA) metabolism and BAs are central regulators of mitochondrial function and intestinal physiology. Perturbed BA circulation and metabolism have been implicated with the pathogenesis of various metabolic diseases, colon cancer and intestinal inflammation (Chen et al., 2019). Primary BAs are generated in the liver, stored in the gall bladder and secreted into the intestinal tract, where they are deconjugated and fermented by the gut microbiota into secondary BAs (Chiang, 2009). BAs mainly function in nutrient absorption, but BAs also serve as potent hormone-like signaling molecules (Sipe et al., 2020). Secondary BAs engage the nuclear receptor and transcriptional regulator farnesoid X receptor (FXR) and the G protein-coupled bile acid receptor 1 (GPBAR1, also known as TGR5) and thereby modulate carbohydrate and lipid metabolism by increasing FAO and OxPhos (Nie et al., 2015). Various molecular regulators are involved in these metabolic adaptations. The epigenetic regulator Sirtuin 1 (SIRT1), the hormone fasting-induced adipose factor (FIAF) and the transcriptional regulators Sterol regulatory element-binding protein 1c (SREBP-1c) and carbohydrate response element binding protein (ChREBP) seem to be central in coordinating the effects of BAs on carbohydrate metabolism (Kuipers et al., 2014; Joyce and Gahan, 2016), whereas PPAR is crucial for coordinating the effects of BAs on fatty acid metabolism (Joyce and Gahan, 2016; Clark and Mach, 2017). In addition to serving as signaling molecules, secondary BAs also modulate mitochondrial biogenesis (Clark and Mach, 2017) and also directly alter mitochondrial function. For instance, deoxycholic acid may alter membrane fluidity and disrupt mitochondrial membrane structure (Sousa et al., 2015). Moreover, hydrophobic BAs induce apoptosis (Faubion et al., 1999) potentially by mPTP formation leading to release of mitochondrial constituents, OxPhos failure and enhanced ROS generation (Gores et al., 1998; Rodrigues et al., 1998; Yerushalmi et al., 2001). This oxidative stress contributes to disease development, for example chronic inflammation, and epidemiological studies suggested that the secondary BAs play a role in the pathogenesis of colorectal cancer (Bayerdörffer et al., 1993; Payne et al., 2008; Nguyen et al., 2018).

Lastly, the microbiota also metabolizes proteins and amino acids, and thereby impacts on intestinal inflammation and carcinogenesis by modulating mitochondrial function. Two amino acids are of particular interest in this context: cysteine and tryptophan. Cysteine is a non-essential proteinogenic amino acid and exerts pleiotropic effects on mitochondrial function (Bonifácio et al., 2021). Cysteine is a component of the antioxidant glutathione, which detoxifies mitochondrial ROS, and is also metabolized into hydrogen sulfide (H_2_S) by both intestinal epithelial cells and to a much greater extend by the microbiota. This microbial H_2_S production is of importance to intestinal health since at low concentrations (nanomolar to low micromolar) H_2_S primes mucosal barrier functions and protective immunity, whereas at high concentrations (high micromolar to millimolar) it promotes intestinal inflammation (Blachier et al., 2019). Mechanistically, H_2_S feeds into the mitochondrial electron transfer chain and mediates persulfidation of ATPase and glycolytic enzymes, thereby stimulating cellular bioenergetics (Bonifácio et al., 2021). Excess H_2_S production by the microbiota, for example by Fusobacterium, associates with intestinal inflammation and cancer (Blachier et al., 2019). In contrast, cysteine deprivation also leads to mitochondrial dysfunction and ultimately mitochondria-dependent cell death, which can be counteracted by deletion or inhibition of fumarate hydratase, a tumor suppressor and TCA cycle component (Gao et al., 2019). Thus, dysregulation of cysteine metabolism impairs mitochondrial function leading to intestinal pathologies.

In contrast to cysteine, tryptophan is an essential amino acid and therefore needs to be taken up from external sources, for example diet or microbial production. Tryptophan attracted significant interest in the past decade as it emerged as key regulator of inflammatory responses and metabolic control (Agus et al., 2018). We will therefore only focus on the microbial contribution and its impact on mitochondrial function. In contrast to humans, various members of the microbiota including *Escherichia coli* are capable of producing tryptophan, which can then be made available to the eukaryotic host. Additionally, the microbiota sheds and produces various ligands engaging the aryl hydrocarbon receptor (AhR), a central regulator of tryptophan metabolism and intestinal immunity that can translocate also into the inner membrane space of mitochondria to bind metabolic stimuli (Hwang et al., 2016; Gao et al., 2018). Tryptophan metabolism occurs via three distinct pathways defined by their characteristic metabolic intermediates (kynurenine, serotonin, and indole) (Agus et al., 2018), that partially take place in mitochondria. Additionally, some microorganisms also possess enzymatic activities to metabolize tryptophan (Kurnasov et al., 2003; Boulangé et al., 2016). Notably, the kynurenine pathway is particularly focused on mitochondria as it yields, among other metabolites, nicotinamide adenine dinucleotide (NAD) and NAD phosphate (NADP), cofactors for redox reactions (Ito et al., 2003; Richard et al., 2009). NAD functions in glycolysis, TCA and OxPhos (Rodriguez Cetina Biefer et al., 2017) and thus drives mitochondrial bioenergetics. NAD also contributes to mitochondrial biogenesis and ROS production through engaging SIRT3 (Duan, 2013; Tullius et al., 2014). This metabolic reprogramming is essential for T cell differentiation and activation and regulating immune responses (Angajala et al., 2018). Similar to immune responses, tryptophan metabolites modulate mitochondrial functions and exert an antiproliferative activity in various cancer cells pointing to a therapeutic potential of tryptophan metabolism in cancer (Walczak et al., 2020).

## Therapeutic Strategies Targeting Microbiota- Mitochondria Interactions

The intestinal microbiota and its metabolites mediate various metabolic, immunomodulatory, and anti-tumorigenic effects that involve tuning mitochondrial function. Alterations in the composition or function of the microbiota can lead to a disruption of homeostasis termed dysbiosis and are associated with various pathologies including metabolic diseases, chronic inflammation and cancer. Therefore, composition of the intestinal microbiota and their metabolite profile may serve as prognostic biomarkers for disease (Aden et al., 2019; Matsushita et al., 2021). Unlike its human host, the composition and function of the microbiota is highly flexible and can be modulated by, for example, antibiotics or nutrition, making it an attractive target for disease therapy and prevention. For example, selective antibiotic treatment with vancomycin removed gram-positive bacteria, which contain the dominant bacteria converting primary to secondary BAs, was necessary to induce natural killer T cell accumulation and to decrease tumor growth (Ma et al., 2018). Alternatively, probiotic supplementation with live bacteria that have specific metabolic features, for example butyrate production, could be used to raise local butyrate levels and thereby restore beneficial mitochondrial bioenergetics and ultimately alleviate chronic inflammation (Hinrichsen et al., 2021). Of course, microbial metabolites could also be supplemented directly for therapeutic purposes. Clinical studies using oral or rectal butyrate supplementation in patients with intestinal inflammation showed promising anti-inflammatory effects (Scheppach et al., 1992; Di Sabatino et al., 2005, 2007) and antitumor therapeutic efficacy in cancer patients (He et al., 2021). One limitation in the therapeutic use of microbial metabolites is that they often have pleiotropic effects. Thus, more specific interventions should be preferable. In the past years many molecular pathways in the communication between microbiota and mitochondria have been revealed that will allow for the development of highly specific interventions. For example, butyrate supplementation also caused gastrointestinal complications including severe diarrhea. With the identification of HK2, mitochondrial respiration and cell death regulation as molecular and subcellular framework mediating some of the beneficial effects of butyrate, clinical trials with specific HK2 inhibitors might allow for improved therapeutic responses while causing fewer complications. Taken together, SCFAs but also other microbial metabolites impact mitochondrial functions and thereby the cellular homeostasis with wide-ranging consequences for metabolism, differentiation, signaling, cell death regulation and ultimately disease susceptibility and pathogenesis. In turn, changes in mitochondrial function can also alter the microbiota and thereby disease development (Schilf et al., 2021) pointing toward a bidirectional communication between microbiota and mitochondria.

## Conclusion

Mitochondria fulfill critical bioenergetic functions that not only coordinate cellular homeostasis, but also enable the regulation of immune cell activation. The microbiota emerged as a central modulator also of mitochondrial function with consequences for host metabolism, immune responses, cell death and proliferation. Dysregulation of these interactions between microbiota and mitochondria are associated with intestinal inflammation and cancer. However, the plasticity of the microbiota along with the identification of molecular pathways in the communication between microbiota and mitochondria now enables the development of microbiota-centered interventions that modulate critical mitochondrial functions and thereby disease susceptibility and treatment. Preclinical and clinical studies are required to unravel the therapeutic potential of microbiota-mitochondria interactions.

## Data Availability Statement

The original contributions presented in the study are included in the article/supplementary material, further inquiries can be directed to the corresponding author/s.

## Author Contributions

FS designed the article concept and obtained funding. SW-S, LJ, and FS designed and prepared the display item. All authors wrote the manuscript. All authors contributed to the article and approved the submitted version.

## Conflict of Interest

The authors declare that the research was conducted in the absence of any commercial or financial relationships that could be construed as a potential conflict of interest.

## Publisher’s Note

All claims expressed in this article are solely those of the authors and do not necessarily represent those of their affiliated organizations, or those of the publisher, the editors and the reviewers. Any product that may be evaluated in this article, or claim that may be made by its manufacturer, is not guaranteed or endorsed by the publisher.
